# Socioeconomic and citizenship inequalities in hospitalisation of the adult population in Italy

**DOI:** 10.1371/journal.pone.0231564

**Published:** 2020-04-23

**Authors:** Alessio Petrelli, Anteo Di Napoli, Elena Demuru, Martina Ventura, Roberto Gnavi, Lidia Di Minco, Cristina Tamburini, Concetta Mirisola, Gabriella Sebastiani

**Affiliations:** 1 National Institute for Health, Migration and Poverty (INMP), Rome, Italy; 2 Epidemiology Unit, ASL TO3, Grugliasco, Turin, Italy; 3 Ministry of Health, Rome, Italy; 4 Italian National Institute of Statistics (Istat), Rome, Italy; Newcastle University, UNITED KINGDOM

## Abstract

**Background:**

Higher levels of hospital admissions among people with lower socioeconomic level, including immigrants, have been observed in developed countries. In Europe, immigrants present a more frequent use of emergency services compared to the native population. The aim of our study was to evaluate the socioeconomic and citizenship differences in the hospitalisation of the adult population in Italy.

**Methods:**

The study was conducted using the database created by the record linkage between the National Health Interview Survey (2005) with the National Hospital Discharge Database (2005–2014). 79,341 individuals aged 18–64 years were included. The outcomes were acute hospital admissions, urgent admissions and length of stay (1–7 days, > = 8 days).

Education level, occupational status, self-perceived economic resources and migratory status were considered as socioeconomic determinants.

A multivariate proportional hazards model for recurrent events was used to estimate the risk of total hospital admissions. Logistic models were used to estimate the risk of urgent hospitalisation as well as of length of stay.

**Results:**

Low education level, the lack of employment and negative self-perceived economic resources were conditions associated with the risk of hospitalisation, a longer hospital stay and greater recourse to urgent hospitalisation. Foreigners had a lower risk of hospitalisation (HR = 0.75; 95% CI:0.68–0.83) but a higher risk of urgent hospitalisation (OR = 1.36; 95% CI:1.18–1.55) and more frequent hospitalisations with a length of stay of at least eight days (OR = 1.19; 95% CI:1.02–1.40).

**Conclusions:**

To improve equity in access, effective primary, secondary and tertiary prevention strategies must be strengthened, as should access to appropriate levels of care.

## Introduction

In Europe, rates and trends of hospitalisation are quite heterogenous, with the highest values in Bulgaria, Germany and Austria [1]. Italy is the most long-lived country in Europe; the life expectancy at birth has increased from 77.9 years in 1995 (males:74.8, females:81.1) to 82.3 in 2015 (males:80.1, females:84.6), though the trend has been heterogenous among the social classes [2]. These results can partially be attributed to the Italian National Health Service, which guarantees to all citizens universal and fair access to healthcare services deemed “essential levels of care”.

Despite this increase in life expectancy, hospitalisation rates in Italy are among the lowest in Europe and continue to decrease, from 172 (*1.000 ab.) in 2010 to 132 (*1.000 ab.) in 2016 [3]. This is due primarily to the effect of healthcare policies aiming at improving organisational appropriateness and to the increase in efficiency margins in resource allocation. However, as such measures concerned primarily those regions with higher debt, they may have led to an indirect rationing of services, thereby not meeting the health needs of the population. The measures introduced have led to a progressive reduction in the number of hospital beds, with the standard defined as 3.7 hospital beds available per 1.000 inhabitants, among the lowest in Europe.

Numerous studies in developed countries have highlighted that hospital admissions are more frequent among people at a low socioeconomic level [4–6]; this has been observed in Italy as well [7]. These findings may be partially explained in terms of health needs: disadvantaged groups have more likely worse health status due to a higher prevalence of multiple chronic diseases [8]. Nevertheless, only a few studies have examined these differences by taking into account direct measures of health needs [9].

Immigrants are a subgroup of the population that is particularly vulnerable to inequalities in health and healthcare. In Europe, immigrants present differences in hospital admissions related to multiple factors, both in terms of the intensity of use–in some cases greater, in other cases lesser–and in terms of access, with a more frequent use of emergency services compared to native population [10].

Despite the fact that strong socioeconomic inequalities in health have been observed in numerous European countries [11], including Italy [12], evidence measured by means of longitudinal population studies is scanty [13]. Further, no study has evaluated inequalities in hospital admissions based on indicators of need.

Our study aimed to evaluate the socioeconomic and citizenship differences in the hospitalisation of the adult population in Italy.

## Methods

The study was conducted using the database created by the longitudinal extension of the national study sample on health status of the National Health Interview Survey (NHIS), conducted by the Italian National Institute of Statistics (Istat) between 2004 and 2005 [14], by means of the deterministic record linkage with the Istat national database of deaths and causes of death [15] and with the hospital discharge database archive of the Ministry of Health [3]. The NHIS was part of the activities included in the 2011–2013 National Statistical Programme (PSN code: IST-02067) approved by the Italian Presidency of the Council of Ministers (decrees dated 31 March 2011 and 20 April 2012). Constructing the database was possible thanks to an agreement signed in 2015 between Istat, the Ministry of Health and the Piemonte Region (PSN code: IST-02566). All data were fully anonymized before we accessed them.

The NHIS was conducted on a sample of 50,474 families and 128,040 individuals; the sample was representative of the resident population of Italy; it provides information on health status, social determinants, behavioural risk factors and use of healthcare services, collected by means of paper-and-pencil interviewing (PAPI) carried out in the months of December 2004, March, June and September 2005, so as to eliminate any seasonal effect on health. The Istat national database of deaths and their causes registers all events in Italy. The hospital discharge database contains information concerning all hospital admissions, including the type of access to hospitalisation, diagnoses, and any surgery and diagnostic/ therapeutic procedures. The linkage key or personal information necessary to reconstruct the database was available for 98.3% of the sample. A sensitivity analysis showed that the distribution of linked and not linked records for the main sociodemographic characteristics and health status variables were not significantly different from each other. The follow-up period was from 01.12.2004 (start date of interviews) to 31.12.2014.

To identify any loss to follow up due to emigration, the archive was linked with data from the Istat Survey on enrolment and CANcellation registries (ISCAN), which include all the enrolments and cancellations from the Italian municipal registries due to residence transfer: 425 individuals left the cohort during the follow-up period due to residence transfer abroad, censoring their follow-up at the data of their transfer abroad.

Our study was conducted on 79,341 individuals aged 18–64 years at the time of the interview. The choice of the lower age limit was motivated by individuals’ having reached a sufficiently established education level, while the upper limit of 64 years was chosen as the resident immigrant population in Italy over the age of 65 years did not exceed 3% at the time of the survey. Further, the study of this age class permitted an evaluation of the differences in hospital admissions in the working age population according to employment status. We selected all acute hospital admissions except for natural births with no complications after delivery (n = 62,753). Admissions with one or more subsequent transfers in the same day (i.e. discharge and admission in the same day) were considered as one single admission, the duration of which was the sum of all days of each admission. Moreover, two additional outcomes were assessed on the subgroup of individuals with at least one hospital admission (n = 30,002): the type of admission (urgent vs non urgent) and length of stay in hospital, categorized in 1–7 days or > = 8 days, using the 75^th^ percentile of the distribution as the cutoff.

Socioeconomic determinants considered were education level (high, medium, low), occupational status (employed, unemployed), self-perceived economic resources (excellent/adequate, scarce/insufficient) and citizenship (Italian, foreign).

At the time of the interview, the number of immigrants resident in Italy was quite low (4%); the proportion of immigrants in the sample was thus low as well, making it impossible to stratify by area of residence.

Education was categorized in three levels: high (secondary school or university degree), medium (middle school), and low (elementary school or none). We considered as potential confounders the following variables: age (18–34, 35–49 and 50–64 years), sex, BMI (obese/not obese, according to the WHO threshold for obesity: 30 kg/m^2^), smoking habit and the presence of at least one chronic disease. The latter factor was defined according to Istat indicators of poor health and potential presence of functional limitations. Specifically, the interviewed was asked to indicate whether, at the time of the interview, he or she had one or more diseases as diagnosed by a physician included in the following list: diabetes, myocardial infarction, angina pectoris, other heart diseases, stroke and cerebral haemorrhage, chronic bronchitis and emphysema, cirrhosis of the liver, malignant tumours (including lymphoma/leukaemia), Parkinson’s disease, Alzheimer’s disease and dementia [16].

As each study subject can potentially experience multiple hospital admissions, the hazard ratios (HR) of hospitalization by socioeconomic determinants were estimated, taking into account potential confounders and effect modifiers, using the Wei, Lin, and Weissfeld method based on the marginal Cox models [17]. Inference of the parameter is based on the robust sandwich covariance matrix estimate to account for the dependence of the multiple failure times. The proportional hazard assumption was assessed for covariates in the Cox model by graphically evaluating the parallelism of “log-log” curves (− log(− log (S(t))) against log(t) for each category of covariates. Moreover, the test based on the scaled Schoenfeld residual was applied [18].

For the purposes of survival analysis, deaths were considered as right-censored events. Person-time was calculated as the difference between the date of the interview and that of the event under study (hospital admissions) or the date of death or emigration. The date of follow-up end was set as 31/12/2014.

To evaluate the effect of socioeconomic determinants on the type of admission (urgent vs non urgent) and length of stay (1-7days, > = 8days), we used Generalized Estimating Equations (GEEs) for binary outcomes in order to take into account the correlations between admissions occurring for the same subject. Lastly, the interactions between citizenship and education level, occupational status and self-perceived economic resources were tested for all the outcomes considered. The goodness of fit of the models was evaluated through the likelihood ratio test.

## Results

We registered in the 10 years of follow up 62,753 hospital admissions in the cohort, with at least one hospitalisation in 37.8% of the sample.

Table 1 shows the prevalence of individuals by number of hospital admissions, stratified by characteristics. The average number of admissions was 0.79. Those with at least one severe chronic condition, those aged 50–64 years, the least educated and obese individuals had the highest mean number of hospitalizations. The prevalence of those who had three or more admissions was significantly higher among individuals aged 50–64, women, individuals with a lower education level, the unemployed, those who declared scarce or insufficient economic resources, Italians, those who lived in southern Italy, those who declared having at least one serious chronic disease, the obese and smokers.

**Table 1:** Distribution of the sample by number of hospital admissions and characteristics investigated.

	Total number of individuals	Mean number of hospital admissions	Individuals by number of hospital admissions								p-value*
			0		1		2		3 or more		
	n		n	%	n	%	n	%	n	%	
Age groups											
18–34	26,482	0.60	17,575	66.4	5,260	19.9	2,133	8.1	1,514	5.7	< 0.001
35–49	28,842	0.64	19,171	66.5	5,714	19.8	2,096	7.3	1,861	6.5	
50–64	24,017	1.18	12,593	52.4	5,184	21.6	2,658	11.1	3,582	14.9	
Sex											
Men	39,181	0.77	25,292	64.6	7,453	19.0	3,004	7.7	3,432	8.8	< 0.001
Women	40,160	0.81	24,047	59.9	8,705	21.7	3,883	9.7	3,525	8.8	
Severe chronic morbidity											
No severe chronic disease	72,999	0.71	46,638	63.9	14,839	20.3	6,043	8.3	5,479	7.5	< 0.001
At least one severe chronic disease	6,342	1.74	2,701	42.6	1,319	20.8	844	13.3	1,478	23.3	
Body mass index											
< 30 kg/m2	72,271	0.75	45,679	63.2	14,638	20.3	6,020	8.3	5,934	8.2	< 0.001
≥ 30 kg/m2	7,070	1.18	3,660	51.8	1,520	21.5	867	12.3	1,023	14.5	
Smoking status											
Never	42,475	0.72	27,074	63.7	8,577	20.2	3,575	8.4	3,249	7.6	< 0.001
Current or former	36,866	0.87	22,265	60.4	7,581	20.6	3,312	9.0	3,708	10.1	
Citizenship											
Italian	76,951	0.80	47,654	61.9	15,735	20.4	6,744	8.8	6,818	8.9	< 0.001
Foreigner	2,390	0.55	1,685	70.5	423	17.7	143	6.0	139	5.8	
Occupational status											
Working	48,384	0.70	31,145	64.4	9,739	20.1	3,925	8.1	3,575	7.4	< 0.001
Not working	30,957	0.93	18,194	58.8	6,419	20.7	2,962	9.6	3,382	10.9	
Education level											
High	34,474	0.65	22,675	65.8	6,819	19.8	2,646	7.7	2,334	6.8	< 0.001
Medium	32,106	0.78	19,952	62.1	6,614	20.6	2,792	8.7	2,748	8.6	
Low	12,761	1.18	6,712	52.6	2,725	21.4	1,449	11.4	1,875	14.7	
Self-perceived economic resources											
Optimal or adequate	56,056	0.74	35,382	63.1	11,498	20.5	4,679	8.3	4,497	8.0	< 0.001
Scarce or insufficient	23,285	0.90	13,957	59.9	4,660	20.0	2,208	9.5	2,460	10.6	
All	79,341	0.79	49,339	62.2	16,158	20.4	6,887	8.7	6,957	8.8	

*A p-value of 0.05 is used as the cutoff for statistical significance.

Individuals who declared scarce or insufficient economic resources, immigrants and those with at least one serious chronic disease had a significantly higher proportion of urgent hospitalisation (Table 2). The percentage of the sample with a length of stay equal to or greater than 8 days was higher among individuals ages 50–64, men, those with a lower education level, the unemployed, those who declared scarce or insufficient economic resources, those who declared having at least one serious chronic disease, the obese and smokers.

**Table 2:** Distribution of hospital admissions by type of admission, length of stay and characteristics investigated.

	Total number of hospital admissions	Type of admission				p-value	Length of stay (days)						p-value*
		Urgent		Non-urgent			mean	std	1–7		8 or more		
	n	n	%	n	%				n	%	n	%	
Age groups													
18–34	16,007	7,933	49.6	8,074	50.4	< 0.001	4.67	5.67	13,746	85.9	2,261	14.1	< 0.001
35–49	18,460	7,211	39.1	11,249	60.9		5.60	7.61	14,589	79.0	3,871	21.0	
50–64	28,286	11,453	40.5	16,833	59.5		6.95	8.45	19,722	69.7	8,564	30.3	
Sex													
Male	30,104	13,057	43.4	17,047	56.6	< 0.001	6.36	8.10	22,200	73.7	7,904	26.3	< 0.001
Female	32,649	13,540	41.5	19,109	58.5		5.62	7.17	25,857	79.2	6,792	20.8	
Severe chronic diseases													
No severe chronic disease	51,737	21,543	41.6	30,194	58.4	< 0.001	5.69	7.41	40,589	78.5	11,148	21.5	< 0.001
At least one severe chronic disease	11,016	5,054	45.9	5,962	54.1		7.32	8.49	7,468	67.8	3,548	32.2	
Body mass index													
< 30 kg/m2	54,393	23,137	42.5	31,256	57.5	0.0478	5.82	7.39	42,155	77.5	12,238	22.5	< 0.001
≥ 30 kg/m2	8,360	3,460	41.4	4,900	58.6		6.95	8.99	5,902	70.6	2,458	29.4	
Smoking status													
Never	32,148	13,044	40.6	17,561	54.6	0.2414	6.19	7.72	24,192	75.3	7,956	24.7	< 0.001
Current or former	30,605	13,553	44.3	18,595	60.8		5.75	7.54	23,865	78.0	6,740	22.0	
Citizenship													
Italian	61,447	25,908	42.2	35,539	57.8	< 0.001	5.97	7.64	47,048	76.6	14,399	23.4	0.5590
Foreign	1,306	689	52.8	617	47.2		5.93	7.67	1,009	77.3	297	22.7	
Occupational status													
Working	33,818	13,924	41.2	19,894	58.8	< 0.001	5.48	7.10	26,925	79.6	6,893	20.4	< 0.001
Not working	28,935	12,673	43.8	16,262	56.2		6.55	8.18	21,132	73.0	7,803	27.0	
Education level													
High	22,570	9,224	40.9	13,346	59.1	< 0.001	5.28	6.87	18,363	81.4	4,207	18.6	< 0.001
Medium	25,123	10,636	42.3	14,487	57.7		5.91	7.72	19,354	77.0	5,769	23.0	
Low	15,060	6,737	44.7	8,323	55.3		7.12	8.42	10,340	68.7	4,720	31.3	
Self-perceived economic resources													
Optimal or adequate	41,713	16,743	40.1	24,970	59.9	< 0.001	5.70	7.30	32,650	78.3	9,063	21.7	< 0.001
Scarce or insufficient	21,040	9,854	46.8	11,186	53.2		6.52	8.23	15,407	73.2	5,633	26.8	
All	62,753	26,597	42.4	36,156	57.6		5.97	7.64	48,057	76.6	14,696	23.4	

*A p-value of 0.05 is used as the cutoff for statistical significance.

The results of multivariate models (Table 3) show that the risk of hospitalisation increased as education level decreased (HR = 1.11 [95% CI:1.07–1.15] and HR = 1.25 [95% CI:1.18–1.31] for medium and for low education level vs. high, respectively), among the unemployed (HR = 1.12; 95% CI:1.08–1.17) and among those who declared that their economic resources were scarce or insufficient (HR = 1.14; 95% CI:1.10–1.19). Low education level, the lack of employment and negative self-perceived economic resources are also conditions associated with a longer hospital stay (OR = 1.25 [95% CI:1.17–1.35], OR = 1.27 [95% CI:1.20–1.35] and OR = 1.23 [95% CI:1.16–1.29], respectively) and greater recourse to urgent hospitalisation (OR = 1.27 [95% CI:1.19–1.36], 1.07 [95% CI:1.01–1.12] and 1.23 [95% CI:1.17–1.29], respectively). Foreigners have a lower risk of hospitalisation (HR = 0.75; 95% CI:0.68–0.83), but a higher risk of urgent hospitalisation (OR = 1.36; 95% CI:1.18–1.55) and more frequent hospitalisations with a length of stay of at least eight days (OR = 1.19; 95% CI:1.02–1.40). Subjects who declared at least one serious chronic disease have a higher risk of hospitalisation (HR = 2.16; 95% CI:2.05–2.28), urgent hospitalisation (OR = 1.25; 95% CI:1.17–1.33) and a longer length of hospital stay (OR = 1.21; 95% CI:1.14–1.29).

**Table 3:** Results of multivariate statistical models for hospital admissions (HR and 95%CI), type of admission and length of stay (OR and 95%CI).

	Hospital admissions				Type of admission (urgent vs non urgent)				Length of stay (> = 8 vs 1–7)			
	Individuals n = 79,341				Hospital admissions n = 62,753							
	HR*	95% CI			OR*	95% CI			OR*	95% CI		
Education level												
High (ref.)	1.00				1.00				1.00			
Medium	1.11	1.07	to	1.15	1.06	1.01	to	1.12	1.10	1.04	to	1.17
Low	1.25	1.18	to	1.31	1.27	1.19	to	1.36	1.25	1.17	to	1.35
Occupational status												
Working (ref.)	1.00				1.00				1.00			
Not working	1.12	1.08	to	1.17	1.07	1.01	to	1.12	1.27	1.20	to	1.35
Self-perceived economic resources												
Optimal or adequate (ref.)	1.00				1.00				1.00			
Scarce or insufficient	1.14	1.10	to	1.19	1.23	1.17	to	1.29	1.23	1.16	to	1.29
Citizenship												
Italian (ref.)	1.00				1.00				1.00			
Foreigner	0.75	0.68	to	0.83	1.36	1.18	to	1.55	1.19	1.02	to	1.40
Severe chronic morbidity												
No severe chronic disease (ref.)	1.00				1.00				1.00			
At least one severe chronic disease	2.16	2.05	to	2.28	1.25	1.17	to	1.33	1.21	1.14	to	1.29

*Adjusted for age, sex, BMI, smoking habit.

The interactions between all the indicators of socioeconomic level (education level, occupational status and self-perceived economic resources) and citizenship did not prove to be statistically significant for any of the three study outcomes.

## Discussion

The results of our study highlight a greater recourse to hospitalisation among the socially more disadvantaged subgroups of the population: those with a lower education level, the unemployed or those who consider their economic resources insufficient. These subjects may have greater need for healthcare due to the systemic conditions generated by the accumulation of disadvantages arising from their greater vulnerability, which could determine worse health status and thus more frequent recourse to hospitalisation [19].

The more disadvantaged individuals also show a greater probability of longer length of hospital stay and of emergency access to hospitalisation. Immigrants show a lower probability of hospitalisation but longer length of stay and greater emergency access to hospitalisation. Moreover, we observed that regarding recourse to hospital admission, foreigners in Italy did not have an increased disadvantage due to education level, based on the results of the interaction test (not shown).

Our results are consistent with those observed in other studies carried out in high-income countries [4, 5, 9, 20–21], where higher hospitalisation rates of more disadvantaged individuals are at least partially explained by more frequent and more serious diseases arising from exposure to unhealthy lifestyles and to settings that put health at greater risk, as well as to limited recourse to primary and to secondary care [22–23].

Our results were obtained by adjusting for the presence of serious chronic conditions and for two risk factors of negative health outcomes: smoking and obesity. However, in the population under study, also factors other than health status may contribute to explaining the higher rate of hospitalisation among the disadvantaged, suggesting inappropriate access to hospital care [24]. More frequent recourse to urgent hospitalisation has also been observed at the international level [25] as well as in Italy [26], underlining how in many healthcare systems, the main doorway to accessing the national health service for immigrants and the more marginalised subgroups of the population is emergency services. As has been observed for the most disadvantaged native Italians, this modality of access to the health service could highlight non optimal primary and secondary care for the immigrant population [24, 27] due to organisational, bureaucratic, cultural and linguistic barriers to access. It is known that promoting access to services for the most disadvantaged classes has positive repercussions on the entire resident population, as in the case of those female cancer screening programmes with higher levels of coverage thanks to the effectiveness in reaching foreign women as well [28]. Removing organisational and bureaucratic barriers, along with linguistic and cultural barriers for immigrants, may promote access to services for the entire population, especially in the more disadvantaged classes. For example, greater flexibility in the office hours of primary care clinics may make it more possible for female immigrants, who often work as domestic workers or as caregivers and have only one free day per week, to access care.

We observed a higher probability of urgent admissions among more disadvantaged people. We can hypothesize that this phenomenon could be associated with reduced recourse to outpatient care [29], determining less attention to one’s health. This can result in delayed diagnosis and in creating more serious conditions of hospitalisation, which in turn could explain the greater frequency of urgent hospital admissions. It has been demonstrated that timely access to primary care is associated with reduced rehospitalisation at 30 days [30] for cardiovascular diseases, the most frequent cause for hospital admission, including among foreigners [31]. Another issue is that more disadvantaged patients refer to specialist care less frequently [32] due to economic barriers or to underestimating one’s health status. Underuse of outpatient care for diagnostic purposes could determine the need to carry out further diagnostic testing during hospitalisation, thus delaying treatment and increasing the length of hospital stay [6, 13].

The lengthier hospital stay among the more disadvantaged, in particular foreigners, could be another consequence of a more limited access to primary healthcare. For example, a patient admitted for stroke who requires rehabilitation may delay acute care discharge because of the reduced possibility of accessing post-acute or community rehabilitation services [33]. Thus physicians may be reluctant to discharge a patient in whom they fear lower compliance with treatment and post-discharge care [34].

However among foreigners the probability of hospitalisation is lower than that of Italians, probably due to the better health status of the immigrant population, as recently confirmed in Italy as well [35]. The so-called "healthy migrant effect" may explain the lower hospitalisation rates in countries such as Italy and Spain, where there is a younger migratory tradition compared to that of native population [36]. A recent study conducted in Italy, however, reports higher levels of hospitalisation among foreigners for stroke, cervical cancer and appendectomy, signalling issues in primary prevention, in diseases with infections contracted in the countries of origin and in hospitalisations at risk of inappropriateness [37]. Unfortunately, due to the limited sample, our data do not allow an assessment of the differences in hospitalisation by groups of causes.

Our study has some original elements. First of all, it is based on a 10-year follow up of a representative sample of the resident population in Italy. The analysis dataset also includes a large set of demographic and socioeconomic covariates, as well as information concerning health status and lifestyle. Thanks to this trove of information, our study is one of the few [9], and the first in Europe, to take into account serious chronic diseases in evaluating the socioeconomic differences in hospital admissions, unlike most population studies, which consider exclusively age due to the unavailability of direct indicators of needs in the sources used [38]. The availability of this information is an added value of our study because we may evaluate factors associated with use of hospital care different from health status.

Our study does, however, have some limitations, including the fact that the low number of hospital admissions made it impossible to conduct any analyses by groups of causes, which would have supported more in-depth interpretations of the phenomena observed. For example, it would have been interesting to measure the impact of avoidable hospital admissions on socioeconomic differences [39] to evaluate what portion of the excess admissions of the socially disadvantaged was due to the appropriateness of hospitalisation. To achieve this, it is necessary to wait for the extension of follow up, which will make it possible to examine a larger number of hospital admissions or, for evaluations concerning immigrants, for the longitudinal extension of the 2012 NHIS survey.

The sociodemographic information used as determinants or confounders in the analyses were collected at baseline, i.e. at the time of the interview. Thus, the assumption that the condition observed at the time of the interview did not change during follow up may be another potential limitation of this study. Finally, employment status may have changed more significantly during the observation period, with an increase in the number of the unemployed, given that this period was characterized by the world economic crisis. Nevertheless, under this hypothesis the bias in the hazard ratios of occupational status would probably be directed towards the null hypothesis, determining an underestimation of the effect.

## Conclusions

This study contributes to documenting inequalities in recourse to hospital admissions by socioeconomic level and citizenship, supported by the availability of information on serious chronic diseases in the population, even if self-reported. Our findings suggest that the more disadvantaged populations, particularly the immigrant population, are admitted to hospital in more serious condition due also to not having accessed services at other levels of care.

On the basis of our results, we think that to improve equity in access, primary, secondary and tertiary prevention strategies should be strengthened, as should access to appropriate levels of care.

The Italian National Health Service is based on universal care and its objective is therefore to guarantee health and healthcare equity. Promoting prevention actions that are more oriented towards equity, along with greater attention to the appropriateness of hospital admissions, should reduce not only health inequalities but also the costs of hospital care [40].
